# High Circulating Caspase-Cleaved Keratin 18 Fragments (M30) Indicate Short-Term Mortality in Critically Ill Patients

**DOI:** 10.1155/2018/8583121

**Published:** 2018-07-08

**Authors:** Alexander Koch, Eray Yagmur, Janine Linka, Fabienne Schumacher, Jan Bruensing, Lukas Buendgens, Ulf Herbers, Ger H. Koek, Ralf Weiskirchen, Christian Trautwein, Frank Tacke

**Affiliations:** ^1^Department of Medicine III, RWTH-University Hospital Aachen, Pauwelsstrasse 30, 52074 Aachen, Germany; ^2^Medical Care Center, Dr. Stein and Colleagues, Mönchengladbach, Germany; ^3^Section of Gastroenterology and Hepatology, Department of Internal Medicine, Maastricht University Medical Centre (MUMC), Maastricht, Netherlands; ^4^Institute of Molecular Pathobiochemistry, Experimental Gene Therapy and Clinical Chemistry, RWTH-University Hospital Aachen, Pauwelsstrasse 30, 52074 Aachen, Germany

## Abstract

Caspase-cleaved fragments of the intermediate filament protein keratin 18 (cytokeratin-18 (CK18)) can be detected in serum as M30 levels and may serve as a circulating biomarker indicating apoptosis of epithelial and parenchymal cells. In order to evaluate M30 as a biomarker in critical illness, we analyzed circulating M30 levels in 243 critically ill patients (156 with sepsis, 87 without sepsis) at admission to the medical intensive care unit (ICU), in comparison to healthy controls (*n* = 32). M30 levels were significantly elevated in ICU patients compared with healthy controls. Circulating M30 was closely associated with disease severity but did not differ between patients with sepsis and ICU patients without sepsis. M30 serum levels were correlated with biomarkers of inflammation, cell injury, renal failure, and liver failure in critically ill patients. Patients that died at the ICU showed increased M30 levels at admission, compared with surviving patients. A similar trend was observed for the overall survival. Regression analyses confirmed that M30 levels are associated with mortality, and patients with M30 levels above 250.8 U/L displayed an excessive short-term mortality. Thus, our data support the utility of circulating levels of the apoptosis-related keratin fragment M30 as a prognostic biomarker at ICU admission.

## 1. Background

Excessive systemic inflammation as a consequence of massive innate immune cell activation is a key characteristic in critically ill patients. This is triggered by infection-related molecules, termed pathogen-associated molecular patterns (PAMPs), and by the release of endogenous immunogenic signals, termed danger-associated molecular patterns (DAMPs) [1]. Activated innate immune cells release abundant inflammatory cytokines (e.g., tumor necrosis factor and interleukins) that initiate inflammatory and also cell death signaling cascades in immune cells and parenchymal cells [2]. While excessive systemic inflammation is well recognized as a major driver of multiple organ failure and mortality in critical illness [3], the resulting mode of cell death, such as necrosis, apoptosis, necroptosis, pyroptosis, or ferroptosis, is incompletely understood [4]. Nonetheless, targeting apoptotic pathways has been suggested as a potential novel therapeutic approach in sepsis therapy [5–7].

The systemic release of caspase-cleaved fragments of keratin 18 (K18), a major type I intermediate filament protein of the cytoskeleton, has been identified as a specific biomarker reflecting apoptotic cell death. K18 occurs in all single-layer epithelial cells such as the gut, urinary tract, or respiratory tract and in parenchymal cells like hepatocytes or cholangiocytes [8]. The caspase-cleaved K18 fragment exposes a circulating neoepitope that is recognized by the specific monoclonal antibody M30, which has been widely described as a valid biomarker in the context of acute and chronic liver diseases [8, 9]. Few data exist on M30 levels in critical illness. Four independent observational studies in patients with sepsis, two of them conducted in small patient cohorts, reported consistently elevated M30 levels compared with controls as well as an association with adverse clinical outcome [10–13]. However, it is currently less well defined if the clinical utility and prognostic relevance of M30 as a biomarker can be extrapolated to heterogeneous cohorts of critically ill patients, in which not only infectious threats (PAMPs) but also sterile inflammation (DAMPs) trigger cell death mechanisms [1]. We therefore studied circulating M30 in a large cohort of 243 critically ill patients at a medical intensive care unit (ICU), in comparison to healthy controls, and assessed its potential as a diagnostic and prognostic biomarker in medical ICU patients.

## 2. Methods

### 2.1. Study Design and Patient Characteristics

In our single-center prospective observational trial, we serially gathered data and blood samples of consecutive patients upon admission to the medical ICU in the University Hospital Aachen, Germany. We excluded patients who had an elective procedure or were admitted for postinterventional observational stays [14]. The study protocol was approved by the local ethics committee and undertaken in accordance with the ethical standards laid down in the Declaration of Helsinki (ethics committee of the University Hospital Aachen, RWTH Aachen University, Aachen, Germany, reference number EK 150/06). We secured written informed consent from the patient, the spouse, or the legal guardian according to the German civil code BGB §1896. The patients were categorized as sepsis and nonsepsis according to the Third International Consensus Definitions for Sepsis and Septic Shock (Sepsis-3) [15] and were treated following the current guidelines for treatment of sepsis (Surviving Sepsis Campaign) [16]. As a control cohort, we included healthy blood donors with normal blood count, normal values of liver enzymes, and a negative serology for viral hepatitis and HIV.

To assess the patients' outcome, we defined the ICU mortality as well as overall mortality, which was based on contacting the patients, relatives, and/or their general practitioners in approximately 6-month intervals after discharge from hospital for two years [17]. Short-term mortality was defined as mortality within 30 days after admission to the ICU, independent from whether the patients died at the ICU, in the hospital, or outside the hospital.

### 2.2. M30 Measurements

We obtained blood samples immediately at the time of admission to avoid influence of any therapeutic procedures. After centrifugation, serum was stored at −80°C. M30 was analyzed with a commercial enzyme immunoassay (M30 Apoptosense® ELISA, cat. number 10011, TECOmedical Group, Sissach, Germany).

### 2.3. Statistical Analysis

Because most samples were not normally distributed, the Mann–Whitney *U* test was applied to test for statistically significant differences between two groups. Correlations were assessed by Spearman's rank correlation method. All values, including outside values as well as far-out values, were included. *p* values less than 0.05 were considered as statistically significant.

The Cox regression model was employed to evaluate the prognostic value of M30 on the outcome. Furthermore, the survival was assessed using Kaplan-Meier analysis with a M30 cut-off level that had been calculated via the Youden Index [18]. All analyses were conducted using IBM SPSS Statistics (SPSS; Chicago, Illinois).

## 3. Results

### 3.1. M30 Levels Are Significantly Elevated in ICU Patients Compared with Healthy Controls but Are Not Related to Sepsis

**Figure 1:**
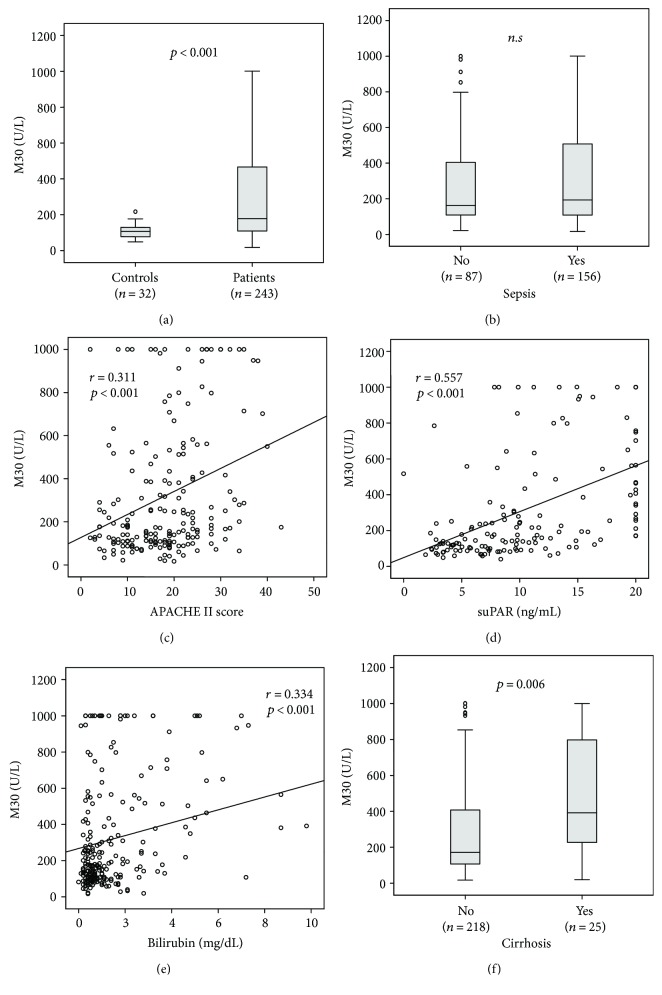
Serum M30 levels in critically ill patients. (a) Serum levels of M30, at the time of admission to the ICU, were significantly higher in critically ill patients than in healthy controls (*p* < 0.001; *U* test). (b) M30 levels did not differ between ICU patients with or without sepsis. (c-d) M30 levels correlated with disease severity, as assessed by the APACHE II score (c) or serum concentrations of soluble urokinase plasminogen activator receptor (suPAR, d). (e-f) M30 levels in critically ill patients correlated with serum bilirubin (e) and were particularly elevated in ICU patients with liver cirrhosis (f).

Circulating M30 levels were significantly increased in critically ill patients (*n* = 243, median 178.3 U/L, range 16.7–1001 U/L, Table 1) compared to the healthy control group (*n* = 32, median 107.1 U/L, range 48.3–217.1 U/L, *p* < 0.001, Figure 1(a)). A total of *n* = 156 patients fulfilled the sepsis criteria, mostly with a pulmonary focus (*n* = 84). Nonsepsis patients (*n* = 87) were admitted due to cardiopulmonary disorders (*n* = 29), decompensated liver cirrhosis (*n* = 13), acute pancreatitis (*n* = 11), and other conditions (Table 2). M30 levels did not differ between patients with sepsis (median 161.8 U/L, range 21.6–1000 U/L) and critical illness without sepsis (median 193.6 U/L, range 16.7–1001 U/L, Table 1, Figure 1(b)).

### 3.2. M30 Serum Concentrations Are Associated with Disease Severity

To identify critical factors that impact M30 levels, we conducted correlation analyses with scoring systems for disease severity as well as with clinically established laboratory parameters representing disease severity (Table 3). We observed significant correlations between circulating M30 levels at admission and clinical scores for disease severity such as the Acute Physiology And Chronic Health II (APACHE II) (*r* = 0.311, *p* < 0.001, Figure 1(c)), Sequential Organ Failure Assessment (SOFA), or Simplified Acute Physiology Score 2 (SAPS2) (Table 3). A positive association was also observed between M30 levels and soluble urokinase plasminogen activator receptor (suPAR, Figure 1(d)), which had been identified as a marker of systemic inflammation and poor prognosis in critically ill patients [19].

### 3.3. M30 Levels Are Correlated with Biomarkers of Liver Failure, Renal Failure, Inflammation, and Cell Injury in Critically Ill Patients

Furthermore, we found strong correlations between M30 levels and biomarkers that reflect hepatic and renal dysfunction (Table 3). More precisely, circulating M30 levels correlated with markers indicating the hepatic biosynthetic capacity (e.g., international normalized ratio (INR), antithrombin III, and pseudocholinesterase), parenchymal damage (e.g., glutamate dehydrogenase (GLDH), aspartate transaminase, and alanine transaminase) and parameters indicating cholestasis (e.g., gamma-glutamyltransferase, alkaline phosphatase, and bilirubin, Figure 1(e)) as well as markers tracing renal dysfunction (e.g., creatinine, cystatin C, and glomerular filtration rate). In addition, circulating M30 levels showed to correlate with parameters of systemic inflammation (e.g., tumor necrosis factor, interleukins 6 and 10, and procalcitonin) and to the general cell injury marker lactate dehydrogenase (LDH, *r* = 0.299, *p* < 0.001).

### 3.4. Patients with Underlying Liver Cirrhosis Have Significantly Elevated M30 Levels

M30 was indicated to be increased in patients with hepatic dysfunction [11, 20]. In accordance, patients admitted to the ICU with liver cirrhosis (*n* = 25, median 391.4 U/L, range 19.4–1000) had significantly increased M30 levels compared to critically ill patients without cirrhosis (*n* = 218, median 171 U/L, range 16.7–1001, *p* = 0.006, Figure 1(f)). Although M30 levels had been specifically linked to nonalcoholic steatohepatitis [9], M30 serum concentrations in our study were not associated with obesity (M30 in patients with BMI > 30 kg/m^2^ median 155.7 U/L versus BMI < 30 kg/m^2^ median 177.8 U/L, not significant).

### 3.5. High M30 Serum Concentrations Are Associated with Excessive Short-Term Mortality

**Figure 2:**
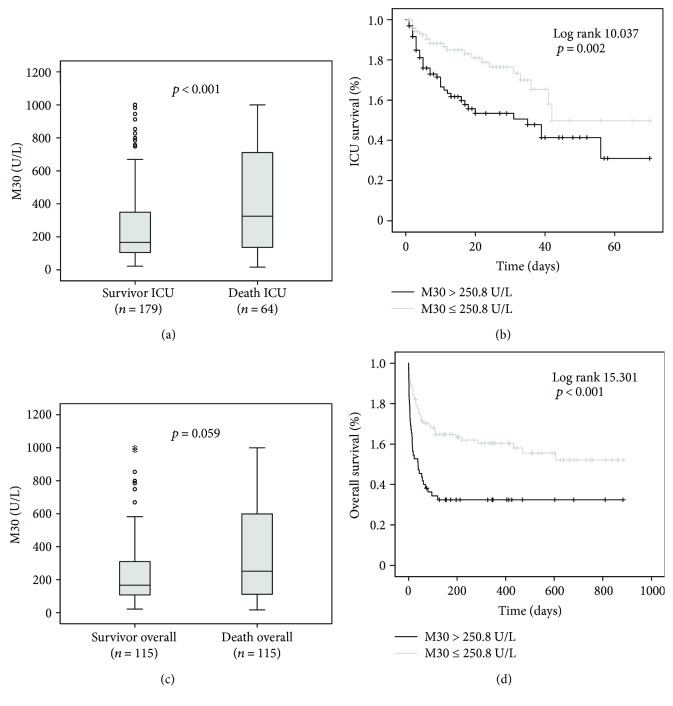
Prediction of mortality by M30 serum levels. (a) Patients that died during the course of ICU treatment had significantly higher serum M30 levels on ICU admission than survivors (*p* < 0.001). (b) On Kaplan-Meier survival curve analysis, ICU patients with M30 levels above 250.8 U/L had increased ICU mortality. (c) Patients that died during the total observation period displayed a trend towards higher serum M30 levels at admission to the ICU than survivors (*p* = 0.059). (d) On Kaplan-Meier survival curve analysis, ICU patients with M30 levels above 250.8 U/L had increased overall mortality, which was apparent especially during the first 30 days after admission. The symbols “o” and “∗” indicate outliers.

We found increased M30 levels at admission in those patients who died at the ICU (*n* = 64, median 324.9 U/L, range 16.7–1000 U/L), compared with surviving patients (*n* = 179, median 166.5 U/L, range 21.6–1001 U/L, *p* < 0.001; Figure 2(a)). Using Cox regression analysis, high M30 levels significantly predicted ICU mortality (*p* = 0.005). Kaplan-Meyer curves were generated by applying the optimal cut-off value (M30 of 250.8 U/L) for the best ratio of sensitivity and specificity for mortality using the Youden index, displaying the prognostic value of high M30 levels for short-term mortality (Figure 2(b)). We detected a trend but no significant difference in M30 levels regarding the overall mortality (nonsurvivors *n* = 115, median 251 U/L, range 16.7–1000 U/L versus survivors *n* = 115, median 166.5 U/L, range 21.6–1001 U/L, *p* = 0.059; Figure 2(c)). However, Cox regression analysis remained significant for predicting overall survival as well (*p* = 0.004), and Kaplan-Meier curves displayed a separation between patients with high versus low M30 levels in their overall survival (Figure 2(d)). The validity and performance of M30 as a biomarker to predict ICU or overall survival in critically ill patients are summarized in Table 4.

As visible from the Kaplan-Meier curve analyses, the majority of deaths that separated patients with high from low M30 levels occurred within the first 30 days. In fact, patients that died within the first 30 days had significantly higher M30 levels (*n* = 74, median 294.6 U/L, range 16.7–1000 U/L) than patients that survived (*n* = 169, median 166.5 U/L, range 21.6–1001 U/L, *p* = 0.001). This difference remained significant at later time points (e.g., 60 days, 90 days, 180 days, and 360 days mortality) but was mainly driven by the difference within the first 30 days (detailed data not shown). In addition, this difference regarding M30 levels and 30 days mortality was significant also in the subgroup of sepsis patients (*p* = 0.007), while the smaller subgroup of nonsepsis patients showed a clear trend towards higher M30 levels in patients that died within 30 days after ICU admission (*p* = 0.066).

## 4. Discussion

During the apoptotic mode of cell death, caspases, intracellular proteases that cleave aspartate residues, become activated either via the intrinsic (mitochondrial release of cytochrome C) or the extrinsic (inflammatory cytokines and death receptors) pathway. The caspases 3, 7, and 9 mediate the early cleavage of the intermediate filament protein cytokeratin 18 in position 396DALD-S, which results in a neoepitope formation that can be detected in serum by the M30 antibody [8]. Our study revealed significantly elevated M30 serum levels in a heterogeneous cohort of critically ill medical patients in close association with disease severity, indicating that apoptosis is a common feature in critical illness and impacts prognosis. As expected, M30 levels correlated with biomarkers reflecting systemic inflammation, supporting that excessive innate immune cell activation is a main driver of programmed cell death. Our study clearly demonstrates that the serum apoptosis marker M30 is independent from the concomitant presence of sepsis. This contrasts previous reports from ICU patients, which have uniformly linked elevated M30 to the clinical course of sepsis [10–13]. Independent from an infectious or noninfectious origin of critical illness, M30 levels correlated with markers of organ dysfunction and disease severity in our study. While these data suggest that hepatocyte apoptosis contributes to circulating M30 in critical illness [2], additional cellular sources might include other epithelial cells, the kidney [21, 22], or the gastrointestinal epithelium [23]. As demonstrated in mouse models [24], a substantial quantity of apoptosis in critical illness proceeds in the gut [25], supporting a role of the gut in critical illness by translocation of bacteria through a disrupted intestinal epithelial barrier, alteration of intestinal immune tissue, and changes in intestinal microflora [23, 26]. Altogether, our data emphasize that not only hypoxia- or hypoperfusion-triggered necroses determine the prognosis in critically ill patients but also apoptotic pathways that are being boosted by systemic inflammation.

Notably, M30 levels are already quite widely used as a biomarker in chronic liver disorders, especially in nonalcoholic fatty liver disease [8]. In our study, we observed the highest M30 levels in patients with hepatic dysfunction and/or liver cirrhosis. This is well in line with previous studies reporting massively elevated M30 levels in patients with acute liver failure [27], acute-on-chronic liver failure [20], and decompensated liver cirrhosis [9]. These associations emphasize the crucial role of the hepatic function for the prognosis in critical illness, as the assessment of liver failure is already included in several prognostic ICU scores such as the SOFA score [2].

In our study, circulating M30 was an early predictor of adverse outcome upon admission of medical patients to the ICU. M30 levels are correlated to disease severity, organ failure, and short-term mortality at the ICU, independent of the presence of sepsis. Our findings indicate a broad clinical relevance of apoptosis in critically ill patients and give impulses for further research. With its strong prognostic value already at ICU admission, M30 is likely to improve risk assessment, if included in novel multimarker panels or clinical scoring systems.

Furthermore, the association between high circulating M30 and increased mortality at the ICU in our study implies that a transient inhibition of apoptotic pathways could potentially reduce the excessive short-term mortality in these patients. While no such clinical trials, stratified by M30 levels, are available in critically ill patients, experimental evidence from animal models suggests that prevention of apoptosis might improve survival in endotoxemia and sepsis [7]. A selective caspase 3 as well as a pan-caspase inhibitor reduced mortality in a mouse model of polymicrobial sepsis, but this effect has been primarily linked to apoptosis of lymphocytes [28]. Similarly, hydrodynamic injection of small interfering RNA (siRNA) against either the Fas death receptor or caspase-8, which effectively targets hepatocytes, reduced mortality in septic mice [29]. On the other hand, apoptosis is a physiological process essential for tissue regeneration as well as a crucial regulator preventing persistent (over-) activation of immune cells, making it challenging to therapeutically interfere with this complex and incompletely understood network [5, 30].

## 5. Conclusions

Our study demonstrated that circulating levels of the apoptosis-related keratin fragment M30 are significantly elevated in critically ill patients as compared with healthy controls, independent of the presence of sepsis. M30 levels are correlated with clinical scoring systems for disease severity as well as biomarkers indicating organ dysfunction and inflammation. The remarkably high levels in patients with cirrhosis and the association with liver function tests indicate that hepatocyte apoptosis might contribute substantially to high circulating M30 in critically ill patients. M30 levels above 250.8 U/L at admission to the ICU indicate an unfavourable short-term prognosis. Further research may explore how this biomarker could be implemented in a multimarker risk assessment panel at the ICU or help in guiding interventional strategies targeting apoptotic pathways in critical illness.

## Figures and Tables

**Table 1:** Patient characteristics and M30 serum measurements at ICU admission.

Parameter	All patients	Nonsepsis	Sepsis
Number	243	87	156
Sex (male/female)	154/89	55/32	99/57
Age median (range) (years)	64 (18–90)	61 (18–85)	65 (21–90)
APACHE II score median (range)	18 (2–43)	17 (2–34)	19 (3–43)
ICU days median (range)	7 (1–70)	5 (1–44)	10 (1–70)
Death during ICU *n* (%)	64 (26%)	15 (17%)	49 (31%)
Death during follow-up (total) *n* (%)	115 (47%)	29 (33%)	86 (55%)
Mechanical ventilation *n* (%)	168 (69%)	55 (63%)	113 (72%)
Preexisting diabetes *n* (%)	73 (30%)	30 (35%)	43 (28%)
Preexisting cirrhosis *n* (%)	25 (10%)	16 (18%)	9 (6%)
BMI median (range) (m^2^/kg)	25.9 (15.3–86.5)	25.4 (15.9–53.3)	26.0 (15.3–86.5)
WBC median (range) (×10^3^/*μ*L)	12.8 (0–208)	11.9 (2.5–27.7)	13.1 (0–208)
CRP median (range) (mg/dL)	93 (0–230)	18 (5–230)	153.3 (0–230)
Procalcitonin median (range) (*μ*g/L)	1.2 (0–207.5)	0.35 (0.03–100)	3.4 (0–207.5)
Creatinine median (range) (mg/dL)	1.3 (0–21.6)	1.0 (0.2–15)	1.6 (0–21.6)
INR median (range)	1.17 (0–133)	1.15 (0.9–6.73)	1.18 (0–133)
M30 median (range) (U/L)	178.3 (16.7–1001)	161.8 (21.6–1000)	193.6 (16.7–1001)

For quantitative variables, median and range (in parenthesis) are given.

**Table 2:** Disease etiology of the study population leading to ICU admission.

	Sepsis	Nonsepsis
	156	87
*Etiology of sepsis critical illness* Site of infection *n* (%)	
Pulmonary	84 (54%)	
Abdominal	27 (17%)	
Urogenital	10 (6%)	
Other	35 (23%)	
*Etiology of nonsepsis critical illness n* (%)		
Cardiopulmonary disorder		29 (33%)
Exacerbated chronic obstructive pulmonary disease		3 (3.5%)
Acute pancreatitis		11 (13%)
Acute liver failure		2 (2%)
Decompensated liver cirrhosis		13 (15%)
Severe gastrointestinal hemorrhage		7 (8%)
Neurological diseases		4 (4.5%)
Intoxication		3 (3.5%)
Ketoacidosis/diabetic coma		5 (6%)
Vasculitis		3 (3.5%)
Nonsepsis other		7 (8%)

**Table 3:** Correlations of clinical scores and laboratory parameters with M30 serum concentrations at admission day (Spearman rank correlation test, only significant results are shown).

Parameters	ICU patients	
	*r*	*p*
*Disease severity*		
APACHE II score	0.311	<0.001
SOFA score	0.390	<0.001
SAPS2 score	0.290	0.018
*Inflammation*		
Procalcitonin	0.362	<0.001
suPAR	0.557	<0.001
Interleukin-6	0.163	0.023
TNF	0.441	0.002
Interleukin-10	0.369	<0.001
LDH	0.299	<0.001
*Renal function*		
Creatinine	0.235	<0.001
GFR (creatinine)	−0.224	0.003
Cystatin C	0.292	<0.001
GFR (cystatin C)	−0.285	<0.001
Urea	0.192	0.003
Uric acid	0.158	0.027
*Liver function*		
Protein	−0.269	<0.001
Albumin	−0.212	0.015
Pseudocholinesterase	−0.275	<0.001
Bilirubin	0.334	<0.001
Bilirubin (conjugated)	0.515	<0.001
Gamma GT	0.337	<0.001
Alkaline phosphatase	0.300	<0.001
AST	0.427	<0.001
ALT	0.358	<0.001
GLDH	0.466	<0.001
INR	0.402	<0.001
Prothrombin time	−0.391	<0.001
aPTT	0.389	<0.001
D-dimers	0.498	<0.001
Antithrombin III	−0.420	<0.001
Platelets	−0.185	0.004

Nonsignificant correlations were noted for M30 levels with blood count, sodium, potassium, magnesium, amylase, lipase, creatine kinase, C-reactive protein, thyroid stimulating hormone, vitamin D, parameters of mechanical ventilation, and central venous pressure.

**Table 4:** Serum M30 performance as a biomarker to predict ICU or overall mortality.

	ICU mortality	Overall mortality
M30 (U/L) optimal cut-off	250.8	250.8
Sensitivity	0.61	0.54
Specificity	0.68	0.70
Positive predictive value	0.41	0.63
Negative predictive value	0.83	0.59
Youden index	0.29	0.21
LHR+	1.92	1.82
LHR−	0.57	0.65
Diagnostic odds ratio	3.34	2.79

LHR: likelihood ratio.

## Data Availability

All data are available upon request to the corresponding author, except for data that could possibly link individual patients to the experimental measurements.
